# *Mycobacterium tuberculosis* Rv0309 Dampens the Inflammatory Response and Enhances Mycobacterial Survival

**DOI:** 10.3389/fimmu.2022.829410

**Published:** 2022-02-24

**Authors:** Yongchong Peng, Xiaojie Zhu, Lin Gao, Jieru Wang, Han Liu, Tingting Zhu, Yifan Zhu, Xin Tang, Changmin Hu, Xi Chen, Huanchun Chen, Yingyu Chen, Aizhen Guo

**Affiliations:** ^1^ State Key Laboratory of Agricultural Microbiology, Huazhong Agricultural University, Wuhan, China; ^2^ College of Veterinary Medicine, Huazhong Agricultural University, Wuhan, China; ^3^ National Animal Tuberculosis Para-Reference Laboratory, Huazhong Agricultural University, Wuhan, China; ^4^ Key Laboratory of Development of Veterinary Diagnostic Products, Huazhong Agriculture University, Wuhan, China; ^5^ Hubei International Scientific and Technological Cooperation Base of Veterinary Epidemiology, Huazhong Agricultural University, Wuhan, China; ^6^ International Research Center for Animal Disease, Huazhong Agricultural University, Wuhan, China

**Keywords:** *mycobacterium tuberculosis*, Rv0309, BCG_RS01790, *mycobacterium bovis* BCG, inflammation, pathogenesis

## Abstract

To reveal functions of novel *Mycobacterium tuberculosis* (*M. tb*) proteins responsible for modulating host innate immunity is essential to elucidation of mycobacterial pathogenesis. In this study, we aimed to identify the role of a putative protein Rv0309 encoded within RD8 of *M. tb* genome in inhibiting the host inflammatory response and the underlying mechanism, using *in-vitro* and *in-vivo* experiments. A recombinant *M. smegmatis* strain Ms_rv0309 expressing Rv0309 and a mutant Bacillus Calmette-Guérin (BCG)ΔRS01790 strain with deletion of BCG_RS01790, 100% homologue of Rv0309 in BCG, were constructed. Rv0309 was found to localize in the cell wall and be able to decrease cell wall permeability. Purified recombinant rRv0309 protein inhibited lipopolysaccharide-induced IL-6 release in RAW264.7 cells. BCG_RS01790 in BCG or Rv0309 in Ms_rv0309 strain greatly inhibited production of IL-6, IL-1β, and TNF-α in RAW264.7 cells. Similarly, BCGΔRS01790 strongly induced expression of these cytokines compared with wild-type BCG and complement strain, cBCGΔRS01790::RS01790. Further BCG_RS01790 or Rv0309 suppressed cytokine production through NF-κB p65/IκBα and MAPK ERK/JNK signaling. Importantly, BCG_RS01790 in BCG and Rv0309 in Ms_rv0309 strain enhanced mycobacterial survival in macrophages. Mice infected with BCGΔRS01790 exhibited high levels of IFN-γ, TNF-α and IL-1β, and large numbers of neutrophils and lymphocytes in the early stage, and minimal lung bacterial load and inflammatory damage in late stage of the experiment. In conclusion, the cell wall protein Rv0309 or BCG_RS01790 enhanced mycobacterial intracellular survival after infection likely through inhibition of the pro-inflammatory response and decrease of bacterial cell wall permeability, thereby contributing to mycobacterial pathogenesis.

## Introduction

Tuberculosis (TB), mainly caused by *Mycobacterium tuberculosis* (*M. tb*), is a leading cause of human death from a single infectious agent for a long time. Despite the continuous efforts made under the Stop-TB and End-TB strategies initiated by the World Health Organization, TB has led to approximately 10 million new cases and 1.51 million deaths in 2021 (1). *M. tb* is considered a highly successful intracellular bacterium that subverts host immune responses for long-term persistence (2). The TB epidemic is further exacerbated by risk factors such as the variable efficacy of the only available attenuated live vaccine derived from *Mycobacterium bovis* (*M. bovis*), Bacillus Calmette-Guérin (BCG), the emergence of multi-drug-resistant strains, and the increased risk of co-infection with HIV (3).

Pathogenic mycobacteria have evolved sophisticated strategies to subvert signaling pathways that regulate innate immune responses in the hosts. For example, mycobacterial effector proteins, including secreted and cell surface-associated proteins, suppress the activation of the NF-κB and MAPK signaling pathways in macrophages, allowing mycobacteria to persist within the hostile macrophage environment (4, 5). Some common proteins between pathogenic mycobacteria and BCG could contribute to pathogenesis. PtpA can inhibit the host’s nature immune response during *M. tb* infection (6). Cell wall-surface proteins such as fibronectin-binding protein A (FbpA) and Rv0246c enhance intracellular mycobacterial survival by suppressing host inflammatory cytokine production (7, 8).

On the other hand, the proteins encoded by genes in the 16 genomic regions of difference (RD) 1–16 between pathogenic *M. tb* or *M. bovis* and attenuated BCG strains are of most concern (9). The main reason for BCG attenuation is the deletion of the RD1 locus, which is missed in all BCG daughter strains, and the loss of RD1 locus abrogates ESX-1-dependent secretion (10–12). For example, ESAT6, a secreted RD1 protein, suppresses inflammatory reactions in macrophages (13, 14). However, several additional reports indicate that ESAT6 can trigger innate immune responses and activate both Th1 and Th17 responses (15, 16). In addition, the genetic changes at uncovered RDs include single nucleotide polymorphisms (SNPs), insertion sequences (IS6110), deletions, and tandem duplications (9, 11, 17–20). For instance, the identification of RvD1 and RvD2 as deletions from the *M. tb* H37Rv rather than *M. bovis* BCG indicates that the deletion process of a gene is not ‘one-sided’, with information loss occurring in both human and bovine strains (21). BCG Mexico 1931 lacks one copy of IS6110 and N-RD18 while containing three new RDs, which are designated as (RDMex01) 53, (RDMex02) 655, and (REDMex03) 2,847 bp long, and 55 SNPs representing non-synonymous mutations compared to BCG Tokyo and BCG Pasteur (22). Although numerous *M. tb* effectors have been identified, the mechanisms by which they interfere with the host’s innate immune system remain largely unclear. Further elucidation of these mechanisms will help to reveal *M. tb* pathogenesis (23).

Rv0309, a conserved hypothetical RD8 protein localized in the cell wall and encoded within RD8 of *M. tb* genome has been identified as a novel fibronectin-binding adhesin, containing genetic diversity in diversifying selection to evade host immunity (20, 24–26). Rv0309 is present in *M. tb* and most *M. bovis* BCG strains such as the Pasteur strain but is absent in the BCG-Frappier and Connaught strains (9). The rv0309 gene in *M. tb* shares 100% identity with BCG_RS01790 in *M. bovis* BCG Pasteur strain, while only 73% identity with MSMEG_0635 in *M. smegmatis* mc^2^155. In this study, we aimed to identify the role of this putative protein Rv0309 in inhibiting the host inflammatory response as well as the underlying mechanism, using *in vitro* and *in vivo* experiments. As a result, we found that Rv0309 suppressed pro-inflammatory cytokine production *in vitro* and *in vivo* and enhanced intracellular mycobacterial survival *in vitro* and the lung bacterial load and lung damage in mice after infection.

## Materials and Methods

### Ethics Statement

Rv0309 antiserum development in mice and artificial infection were performed strictly according to the Guidance for the Use and Care of Laboratory Animals, Hubei Province, China. The protocols were approved by the Ethics Committee of Huazhong Agricultural University (protocol no. HZAUMO-2018-027).

### Bacteria and Cell Culture

*M. smegmatis* mc^2^155 (NC_008596.1) and *M. bovis* BCG-Pasteur (ATCC:35734) were a gift from Professor Luiz Bermudez from Oregon State University. All strains were cultured in Middlebrook 7H9 broth (BD, MD, USA) containing 0.5% glycerol (Sigma, MO, USA), 10% oleic acid-albumin-dextrose-catalase (OADC) and (BD) 0.05% Tween 80 (Sigma) or on Middlebrook 7H11 agar plates (BD, MD, USA) containing 0.5% glycerol (Sigma) and 10% OADC (BD). Before infection, optical densities at 600 nm (OD_600_) of bacterial cultures were adjusted to the required multiplicity of infection (MOI) referring to the standard turbidimetric card. Then, the cultures were centrifuged at 3,000 × *g* for 10 min. The precipitated bacteria were resuspended in a medium and dispersed by passage through an insulin syringe. Next, 50 μL of 10-fold serially diluted bacterial suspension was plated onto Middlebrook 7H11 agar (BD) to count viable bacteria (colony-forming units, CFUs).

RAW264.7 cells were cultured in Dulbecco’s modified Eagle’s medium (Gibco, NY, USA) containing 10% (v/v) fetal bovine serum (Gibco) and 100 mg/mL streptomycin and 100 IU/mL penicillin at 37°C in an atmosphere of 5% CO_2_.

### Antibodies

Anti-β-actin antibody (IgG) (60008-1-lg, ProteinTech, IL, USA) was obtained from ProteinTech. Rabbit monoclonal antibodies to JNK2 (56G8) (#9258), phospho-SAPK/JNK (Thr183/Tyr185) (98F2) (#4671), phospho-NF-κB p65 (Ser536) (93H1) (#3033), p38 MAPK (#9212), phospho-p38 MAPK (Thr180/Tyr182) (#9211), p44/42 MAPK (Erk1/2) (137F5) (#4695), and phospho-p44/42 MAPK (Erk1/2) (Thr202/Tyr204) (D13.14.4E) XP^®^ (#4370) and mouse monoclonal antibodies to NF-κB subunit p65 (L8F6) (#6956), NF-κB IκBα (L35A5) (#4814), and phospho-NF-κB IκBα (Ser32/36) (5A5) (#9246) were purchased from Cell Signaling Technology (Cell Signaling Technology, MA, USA).

### *In Silico* Identification and Sequence Analysis of Rv0309

The Aliphatic index and grand average of hydropathicity index (GRAVY) value of the Rv0309 were evaluated using ProtParam (https://web.expasy.org/protparam/) (27). Transmembrane structure forecasting was performed at TMHMM web server (http://www.cbs.dtu.dk/services/TMHMM/) (28). Protein homologs to Rv0309 in mycobacterium were identified with Mycobrowser (https://mycobrowser.epfl.ch) (29). Homologous sequence alignment was conducted online with ESPript web server (https://espript.ibcp.fr/ESPript/cgi-bin/ESPript.cgi) and Clustal Omega (https://www.ebi.ac.uk/Tools/msa/clustalo/) (30, 31). To validate the presence of a conserved domain in the Rv0309 and its homologs, the resulting six protein sequences were subjected to analysis using the NCBI CDD search (https://www.ncbi.nlm.nih.gov/Structure/cdd/wrpsb.cgi) (32). The conserved motifs in these protein sequences were examined by the MEME suite motif search tool (http://memesuite.org/) (33).

### Construction of *M. smegmatis* Containing rv0309

The full-length *rv0309* gene (gene ID: 886574, NC_000962.3: 377931-378587) was amplified from *M. tb* H37Rv genomic DNA, using specific primers (**Table 1**). The target gene was cloned into the pMV261 vector to generate pMV261-Rv0309. pMV261-Rv0309 was electroporated into *M. smegmatis* mc^2^155 to generate a recombinant strain, Ms_rv0309. Briefly, mc^2^155 were electroporated in the presence of 2 μg of pMV261-Rv0309 plasmid DNA with a Gene Pulser (Bio-Rad, USA). The conditions of electroporation were 200 μl volume, 2.5 kV, 25 μF and 1000 Ω, with a 0.2-cm-gap electroporation cuvette. *M. smegmatis* transformed with empty pMV261, designated as Ms_Vec, was used as a control. The constructs were confirmed by colony PCR and western blot assay. The sequencing of the colony PCR product was outsourced to TSINGKE Biological Technology (Wuhan, China). A 657-bp band was amplified from the Ms_rv0309 strain. The specific primers listed in **Table 1** were used for identifying the *rv0309* gene. Ms_rv0309 and Ms_Vec were cultured until an OD_600_ of 0.6. The cells were pelleted and resuspended in lysis buffer (0.1 M PBS, 1 mM phenylmethylsulfonyl fluoride) for ultrasonic lysis (250W, 5s on/5s off, lasting for 25 min). The whole- cell lysates were subjected to western blot assay for detecting the expression of Rv0309 using mouse antiserum to rRv0309, which was prepared and stored at our laboratory.

**Table 1:** Primers used for RT-qPCR and the construction of recombinant and mutant strains.

Primers	Sequences (5′–3′)
IL-6-For	TGCCTTCTTGGGACTGAT
IL-6-Rev	CTGGCTTTGTCTTTCTTGTT
TNF-α-For	CGATGAGGTCAATCTGCCCA
TNF-α-Rev	CCAGGTCACTGTCCCAGC
IFN-γ-For	CAGCAACAGCAAGGCGAAAAAGG
IFN-γ-Rev	TTTCCGCTTCCTGAGGCTGGAT
IL-1β-For	TGAAATGCCACCTTTTGACAG
IL-1β-Rev	CCACAGCCACAATGAGTGATAC
β-actin-For	TGCTGTCCCTGTATGCCTCT
β-actin-Rev	GGTCTTTACGGATGTCAACG
Rv0309-For	GGGGATCCATGAGCCGACTCCTAGCTTTGCT
Rv0309-Rev	CGAAGCTTTTAGTGGTGATGGTGATGATGCTTGGCGATCGCGATCACCG
RS01790LYZ	CAACCTGACAGCGTAGGTCA
RS01790RYZ	TGCATGCGCTTGGTGTAGAT
RS01790-KoLFP	TTTTTTTTCCATAAATTGGTGTTTCGCTCGCTTTTGTCG
RS01790-KoLRP	TTTTTTTTCCATTTCTTGGACAGCAAAGCTAGGAGTCGG
RS01790-KoRFP	TTTTTTTTCCATAGATTGGTGCAGATCATCCGTTGGCTG
RS01790-KoRRP	TTTTTTTTCCATCTTTTGGAGCTCGGGTAACAGAACTGC

### Expression and Purification of Rv0309

The Ms_rv0309 strain was cultured in Middlebrook 7H9 broth (BD) containing 10% OADC (Sigma) and 0.05% Tween-80 until an OD_600_ of 0.6. Rv0309 expression was induced in a water bath at 45°C for 1 h. Ms_rv0309 bacteria were centrifuged at 3,000 × *g* for 10 min and subsequently resuspended in phosphate-buffered saline (PBS) and lysed by ultrasonication (300W, 5s on/5s off, lasting for 30 min). Recombinant rRv0309 was purified using Ni-NTA agarose chromatography (Qiagen, Hilden, Germany) and verified using 12% sodium dodecyl sulfate polyacrylamide gel electrophoresis (SDS-PAGE). The purified rRv0309 protein was stored at –80°C until use (6).

### Construction of BCGΔRS01790 Mutant and cBCGΔRS01790::RS01790 Complement Strain

The BCGΔRS01790 mutant was constructed as described previously, with some modification (34). Cosmid p0004s and phAE159 vectors were kindly offered by Professor Jiaoyu Deng from Wuhan Institute of Virology, Chinese Academy of Sciences. The Phasmid harboring allelic exchange substrates (AES) including flanking sequences of BCG_RS01790 was electroporated into *M. smegmatis* mc^2^155 to get the specialized transducing phages (STPs) from transduced *M. smegmatis* and then used STPs to disrupt BCG_RS01790. Briefly, flanking genomic regions of BCG_RS01790, 817 bp upstream and 699 bp downstream, were amplified and inserted into p0004s at the Van91I site to generate p0004s-L+R. The recombinant cosmid phAE159-p0004s-L+R was obtained by ligating p0004s-L+R with phAE159 and was transformed into *Escherichia coli* HB101 through an *in-vitro* λ packaging reaction with commercial MaxPlax Lambda Packaging Extract (Epicentre Biotechnologies, WI, USA). Phasmid DNA was extracted from confirmed hygromycin-resistant *E. coli* transductants using Plasmid Maxi Kit (Omega, United States). The temperature-sensitive shuttle phasmid DNA was transduced into *M. smegmatis* mc^2^155, which was cultured at 30°C to generate transgenic phages. As previously reported, the phage transduction rate was optimal at an MOI of 10 (35). BCG was infected with the recombinant STPs at 37°C to generate BCGΔRS01790 mutant by integrated phage-specific DNA into BCG genome, which was verified with PCR by using the primers RS01790LYZ and RS01790RYZ (**Table 1**) and sequencing. BCGΔRS01790 was distinguished from the wild-type BCG based on hygromycin resistance. In the mutant, the hygromycin resistance cassette replaced the target gene BCG_RS01790 and expected PCR product sizes for WT BCG (~2.5 kb) and BCGΔRS01790 (~5.5 kb) were obtained.

To construct the complement strain cBCGΔRS01790::RS01790, pMV261-rv0309 was electroporated into BCGΔRS01790, and positive colonies were screened on 7H11 plates containing hygromycin (75 μg/mL) and kanamycin (50 μg/mL). Rv0309 expression in the complement strain was confirmed using PCR and immunoblotting. Briefly, BCGΔRS01790, cBCGΔRS01790::RS01790, and WT BCG were cultured until an OD_600_ of 0.6. The cells were pelleted and resuspended in lysis buffer (0.1 M PBS, 1 mM phenylmethylsulfonyl fluoride) for ultrasonic lysis (250W, 5s on/5s off, lasting for 30 min). Then whole cell lysate was subjected to western blot assay for detecting the absence of Rv0309 using mouse antiserum to rRv0309. An anti-GroEL2 antibody prepared in our laboratory was used to detect the cytoplasmic marker protein GroEL2 of BCG.

### *In Vitro* Growth Kinetics of Recombinant BCG and *M. smegmatis* Strains

To examine growth patterns, OD_600_ values of triplicate cultures of BCG strains (WT BCG, BCGΔRS01790, and cBCGΔRS01790::RS01790) and *M. smegmatis* strains (Ms_rv0309 and Ms_Vec) were adjusted to 0.2. Then, OD_600_ values were determined every 3 days for 60 days for BCG and every 4 h for 5 days for *M. smegmatis*. Growth curves were plotted based on the average OD_600_ values.

### Quantitative Evaluation of the Colony Morphology and Scanning Electron Microscopy of the Recombinant Mycobacterial Strains

The pictures of colonies for each strain were taken with VHX-5000 microscope with a super-wide depth of field and the circularity and diameter of 10 independent colonies of each strain were determined using ImageJ software, according to the developer’s instructions (36, 37). Circularity was calculated as 4π × area/perimeter^2^; a value of 1.0 indicates a perfect circle, and a decrease in the circularity value indicates a less circular colony. For scanning electron microscopy (SEM) observation of the bacteria, Ms_rv0309, Ms_Vec, WT BCG, BCGΔRS01790, and cBCGΔRS01790::RS01790 were cultured until an OD_600_ of 0.6. The bacterial pellets were resuspended in 0.1 M phosphate buffer (pH 7.2) after centrifugation, and then put on poly-L-lysine-coated slides (Thermo Fisher Scientific, Rochester, NY). The bacteria were fixed with 2.5% glutaraldehyde (Solarbio, Beijing, China) at 4°C for 2 hours, washed 3 times with 0.1 M phosphate buffer (pH 7.2), and then postfixed with 1% osmium tetroxide (Sigma-Aldrich, MO, USA) for 1 hour at room temperature. After being dehydrated in a graded ethanol series and permuted with 3-methyl butyl acetate, samples were placed in the critical point dryer for drying (Leica EM CPD300, IL, United States). SEM was performed on a VEGA3 TESCAN instrument (Brno-Kohoutovice, Czech Republic) using an accelerating voltage of 20 kV. The bacterial cell length and width of 20 bacteria of each strain were determined using ImageJ software.

### Subcellular Localization of Rv0309/BCG_RS01790 in BCG and Recombinant *M. smegmatis* Strains

The subcellular localization of Rv0309 was determined using previously reported methods (38). Briefly, Ms_rv0309, Ms_Vec, and WT BCG were cultured until an OD_600_ of 0.6. The cells were pelleted and resuspended in lysis buffer (0.1 M PBS, 1 mM phenylmethylsulfonyl fluoride) for ultrasonic lysis (250W, 5s on/5s off, lasting for 30 min). The lysates were centrifuged at 3,000 × *g*, 4°C for 5 min, and the supernatants were ultracentrifuged at 30,000 × *g*, 4°C for 30 min. After ultracentrifugation, the supernatants (cell membrane and cytoplasmic fractions) and pellets (cell wall fraction) were collected separately, and the pellets were resuspended in PBS. Equal amounts of pellets and supernatants were subjected to western blotting to determine Rv0309 expression. An anti-GroEL2 antibody prepared in our laboratory was used to detect the cytoplasmic marker protein GroEL2 of *M. smegmatis* and BCG. An anti-Ag85A antibody, a gift from Professor Xiang Chen from Yangzhou University, was used to detect the cell wall-associated protein Ag85A of BCG (39).

### Permeability Determination of Recombinant Mycobacterial Strains

Since Rv0309/BCG_RS01790 contains a YkuD_like superfamily domain with a L,D-transpeptidase catalytic site which has been shown to be related to the hinge of bacterial cell wall peptidoglycans (26), it was speculated that expression of Rv0309/BCG_RS01790 should change the permeability of the bacterial cell wall. To confirm the effect of Rv0309/BCG_RS01790 on cell wall permeability, the permeability of the recombinant *M. smegmatis* and BCG was determined using a reported method (40). Briefly, Ms_rv0309, Ms_Vec, WT BCG, BCGΔRS01790, and cBCGΔRS01790::RS01790 were cultured with and without the antibiotics hygromycin until an OD_600_ of 0.6 and then, the cultures were centrifuged at 3,000 × *g* for 10 min. The bacterial pellets were washed with PBS containing 0.05% Tween-80 three times and then, the cells were resuspended in uptake buffer (5 mM MgSO_4_, 50 mM KH_2_PO_4_) and diluted to an OD_600_ of 0.5. Glucose solution (25 mM) was added into the suspension, and the mixture was incubated to pre-energize the strains at room temperature (RT) for 5 min. Then, 200 μL of the bacterial solutions were added to a black, clear-bottomed 96-well microplate, and ethidium bromide (EB) was added to each well at a final concentration of 20 μM. The fluorescence intensity of EB was determined at 5-min intervals for 1 h, using a microplate reader (BMG-Labtech, Offenburg, Germany), with excitation and emission wavelengths of 530 nm and 590 nm, respectively.

### Intracellular Survival of BCG and *M. smegmatis* Strains

RAW264.7 cells were seeded in 12-well plates (1 × 10^6^ cells/well) for 12 h before infection. The cells were infected with Ms_rv0309, Ms_Vec, WT BCG, BCGΔRS01790, and cBCGΔRS01790::RS01790 at an MOI of 10:1 for 4 h (defined as –4 h). Then, the infected cells were washed with PBS three times to remove extracellular bacteria (referred to as 0 h). The infected cells were further cultured in complete medium supplemented with 100 μg/mL gentamicin and lysed with 0.025% (v/v) SDS at 0, 2, 4, 8, and 24 h post-infection (hpi) for *M. smegmatis* and at 0, 2, 4, 8, 24, and 48 hpi for BCG strains. After 10-fold serial dilution, the lysates were plated onto 7H11 agar plates containing 10% OADC. Colonies were counted 7 days after plating for *M. smegmatis* strains and 21 days after plating for BCG strains. The CFU/mL was calculated for each strain.

### Cytokine Production Induced by Recombinant BCG, *M. smegmatis* Strains, and Purified rRv0309 Protein in RAW264.7 Cells

RAW264.7 cells were infected with Ms_rv0309, Ms_Vec, BCGΔRS01790, cBCGΔRS01790::RS01790, and WT BCG at an MOI of 10 for quantitative reverse-transcription PCR (RT-qPCR). At indicated time points, total cellular RNA was extracted using TRIzol reagent (Invitrogen, CA, USA) and was reverse-transcribed into cDNA using HiScript Reverse Transcriptase (Vazyme, Nanjing, China). mRNA levels of *IL-1β*, *IL-6*, and *TNF-α* were detected on a ViiA7 Real-time PCR System (Applied Biosystems, CA, USA) using SYBR Green Master Mix (Vazyme) and were quantified using the 2^–ΔΔCt^ method. The primers used are listed in **Table 1**.

To detect the production of IL-6, TNF-α, and IL-1β, culture supernatants of infected macrophages were collected and analyzed using ELISA kits (Neobioscience, Shenzhen, China) according to the manufacturer’s protocol. To investigate the effect of recombinant protein rRv0309 derived from Ms_rv0309 on cytokine production, RAW264.7 cells were stimulated with rRv0309 protein at various concentrations (5, 10, and 50 μg/mL) and LPS (1 μg/mL) for 18 h (13). Then, cell culture supernatants were harvested to detect IL-6 levels using an ELISA kit (Neobioscience). Each sample was analyzed in triplicate.

### Western Blot Assays of Critical Signaling Molecule Expression

RAW264.7 cells were infected with Ms_rv0309, Ms_Vec, BCGΔRS01790, and WT BCG (MOI = 10) and lysed at indicated time points. The lysates were centrifuged at 12,000 × *g*, 4°C for 10 min. Proteins were separated by SDS-PAGE and transferred to polyvinyl difluoride membranes, which were blocked with 5% bovine serum albumin in TBST. The membranes were incubated overnight with antibodies against non-phosphorylated and phosphorylated JNK, ERK1/2, p38, IκBα, and p65. β-Actin was used as a control. Then, the membranes were washed and incubated with horseradish peroxidase (HRP)-conjugated goat anti-rabbit (1:2000) or anti-mouse IgG (1:2000) (SouthernBiotech, AL, USA) at 25°C for 1 h. Protein signals were detected using WesternBright ECL HRP substrate (Advansta, CA, USA) per the manufacturer’s instructions.

### Effect of Cell Signaling Pathway Inhibitors on Cytokine Production

All cell signaling pathway inhibitors were purchased from Sigma-Aldrich (Shanghai, China), dissolved in sterile dimethyl sulphoxide (DMSO) (Sigma-Aldrich, Shanghai, China) and utilized at varying concentrations. The cells were pretreated with 20 mM PDTC (NF-κB inhibitor), 10 mM U0126 (ERK1/2 inhibitor), 20 mM SP600125 (JNK inhibitor), and 20 mM SB202190 (p38 inhibitor) for 1 hour before being infected with BCGΔRS01790. The 0.1% (v/v) DMSO was used as the vehicle control (41). After a 12 infection, the cell supernatants were harvested and subjected to cytokine measurements with the commercial ELISA kits.

### Infection of Mice With WT and Recombinant BCG Strains

In total, 120 6-week-old female C57BL/6 mice weighing 20 ± 2 g were purchased from the Laboratory Animal Center of Huazhong Agricultural University and were randomly divided into four groups of 30 mice each. The mice were infected intratracheally with WT BCG, BCGΔRS01790, or cBCGΔRS01790::RS01790 at a dose of 2 × 10^6^ CFU/mouse in 25 μL of PBS, or mock-infected with an equal amount of PBS. At 0, 2, 4, 8, 16, and 21days post-infection (dpi), five mice were weighed, euthanized, and sampled, and sera were collected to detect cytokine levels.

The lungs were collected for bacterial load enumeration and histopathological and immunohistochemical examinations. For enumeration of the lung bacteria, left lung tissues were homogenized. The homogenates were serially diluted and plated onto 7H11 agar plates. Colonies were counted after incubation at 37°C for 14–21 days. For histopathological and immunohistochemical examinations, right lung lobes were fixed in 10% neutralized formalin, embedded in paraffin, and cut to 4-mm sections. Histopathological analysis was performed through conventional hematoxylin and eosin staining (6). Inflammatory cells, including neutrophils, lymphocytes, and monocytes, were counted manually by an experienced pathologist based on morphological criteria in 10 random fields in one random slide for each lung (37). These were assessed in a blinded fashion by the pathologist. The total numbers of the different cell types were compared between the groups. The levels of IFN-γ, TNF-α, IL-4, and IL-17 in the lung tissues were determined by immunohistochemical staining using relevant antibodies. Positive brown signals were quantified using Image-Pro Plus 6.0 (IPP6) software (Media Cybernetics) in five random fields in one random slide for each lung and were expressed as integrated optical density (IOD) and were compared between the groups (42).

In addition, total RNA was extracted from the spleens using TRIzol reagent (Invitrogen) per the manufacturer’s instructions and used for RT-qPCR.

### Statistical Analysis

All assays were conducted in triplicate, and data are expressed as the mean ± standard error of the mean. All experiments were repeated three times independently. GraphPad Prism 7.0 (La Jolla) was used for statistical analysis. A two-tailed unpaired *t*-test with Welch’s correction was used for comparison of two groups, and one-way or two-way ANOVA followed by the LSD test was used for comparison of multiple groups. Statistical significance is expressed at four levels: not significant (ns, *p* > 0.05), *p < 0.05, **p < 0.01, and ***p < 0.001. Gray-scale values for western blot bands were analyzed using ImageJ (National Institutes of Health).

## Results

### Bioinformatics Analysis of Rv0309

**Figure 1:**
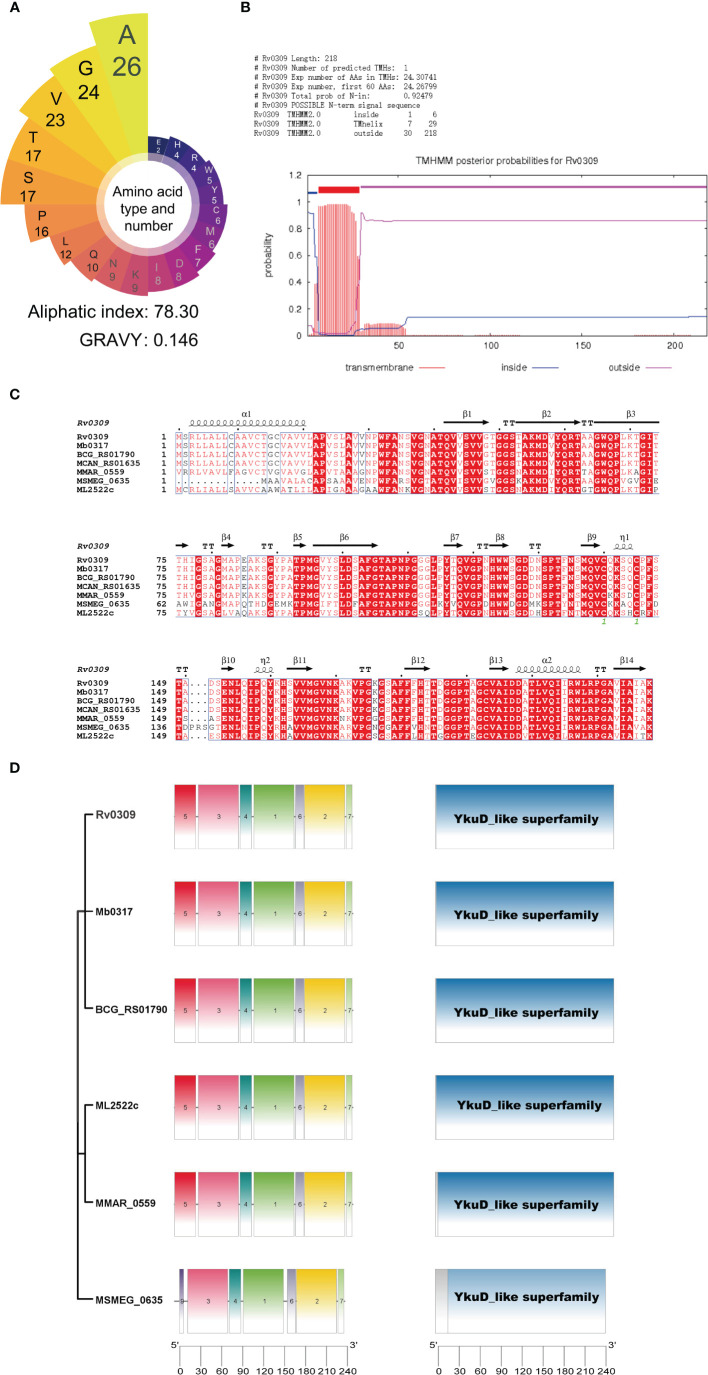
Results of the bioinformatic analysis on Rv0309. **(A)** Amino acid types and number of Rv0309. The grand average of hydropathicity index (GRAVY) values and Aliphatic index of the Rv0309 were determined using ProtParam web server. **(B)** Transmembrane domain prediction by TMHHM2.0, TMHs = 1 indicates that Rv0309 has one transmembrane helix structure. **(C)** Multiple sequence alignment of protein homologs to Rv0309. An alignment of protein homologs was constructed with the Clustal Omega combined with ESPript 3.0. **(D)** Schematic representation of the YkuD_like domain and conserved motifs identified in the Rv0309 protein and its homologs. The NCBI CDD search tool was used to examine the conserved domain in the Rv0309 and its homologs. Conserved motif searches in these protein sequences were conducted with MEME suite motif search tool.

Bioinformatics analysis was used to identify the conservation and function of Rv0309. As shown in **Figure 1A**, Rv0309 protein contained rich aliphatic amino acids (aliphatic index = 78.30) and the grand average of hydropathicity (GRAVY) value of Rv0309 was 0.146, suggesting that Rv0309 had strong lipophilicity, which might be with transmembrane domain residues and/or with the membrane lipids (**Figure 1A**). Furthermore, a transmembrane structure was predicted in Rv0309 (TMHs = 1) (**Figure 1B**), indicating that this protein was a transmembrane protein, this result also suggested that Rv0309 might interact with extracellular substances. Homology analysis showed that Rv0309 was variably conserved in mycobacteria. Compared to Rv0309 in *M. tb*, the Mb_0317 in *M. bovis*, or BCG_RS01790 in BCG have an identity of 100%. However, the homolog MSMEG_0635 in *M. smegmatis*, MMAR_0559 in *M. marinum*, ML2522c in *M. leprae* have the identity of 72%, 78%, 88%, respectively (**Figure 1C**). Conserved domain analysis revealed that these proteins contain a YkuD_like superfamily domain related to mycobacterial cell wall synthesis (**Figure 1D**). Furthermore, Rv0309 and its homologs belonging to YkuD_like superfamily shared multiple conserved motifs with high homology in Mycobacteria (**Figure 1D**), indicating that this protein is vital to the physiological process of Mycobacteria and its potential function is worthy of deep investigation.

### Rv0309 Expression Is Associated With the Colony Morphology

**Figure 2:**
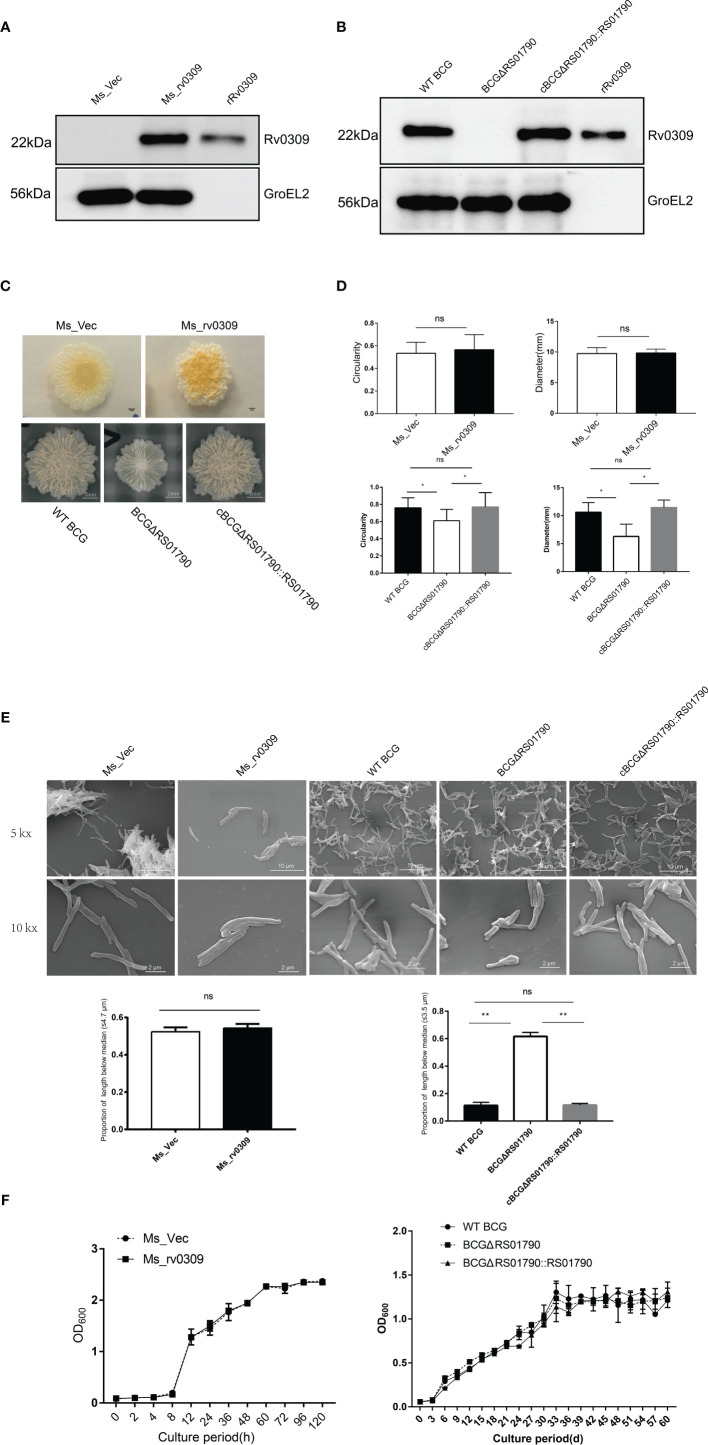
Effect of Rv0309 on the growth rate and morphology of mycobacteria. **(A, B)** *M. tb* Rv0309 expression in *M. smegmatis* and deletion in BCG was determined by western blotting assay using bacterial cell lysates and a rabbit anti-Rv0309 antibody. The purified rRv0309 was used as a positive control, while GroEL2 as an internal reference of *M. smegmatis* and BCG. **(C, D)** The Ms_Vec, Ms_rv0309, WT BCG, BCGΔRS01790, and cBCGΔRS01790::RS01790 strains were cultured on Middlebrook 7H11 agar plates containing 10% OADC for stereomicroscopic colony morphology analysis. For each strain, 10 independent colonies were randomly selected for analysis, and colony circularity and diameters were determined using the ImageJ software. **(E)** Logarithmic growth phase Ms_rv0309, Ms_Vec, WT BCG, BCGΔRS01790, and cBCGΔRS01790::RS01790 were resuspended in 0.1 M phosphate buffer (pH 7.2) after centrifugation, and then put on poly-L-lysine-coated slides. Samples were then processed for SEM. SEM was performed on a VEGA3 TESCAN instrument using an accelerating voltage of 20 kV. The data of length and width for 20 bacteria were determined using ImageJ software. The proportion of bacterial length less than the median was counted, the bacterial length median of BCG and *M. smegmatis* strains was 3.5μm and 4.7μm respectively. **(F)** The Ms_Vec, Ms_rv0309, WT BCG, BCGΔRS01790, and cBCGΔRS01790::RS01790 strains were grown in Middlebrook 7H9 medium containing 0.05% Tween-80 and 10% OADC. OD_600_ values were determined at the indicated time points. A two-tailed unpaired *t*-test and two-way ANOVA were used to determine the statistical significance of differences between different groups (three independent replicates). *p < 0.05 and **p < 0.01 indicate statistically significant differences; while ns indicates no significant difference.

Ms_rv0309 and BCGΔRS01790 strains were confirmed with PCR (**Supplementary Figure 1**). The expression of Rv0309 was identified in Ms_rv0309, WT BCG, and the complement strain cBCGΔRS01790::RS01790, with a band size of 22 kDa using Western blot assay, whereas Rv0309/BCG_ RS01790 was absent in Ms_Vec and BCGΔRS01790 mutant (**Figure 2A, B**). The effect of Rv0309 on colony morphology was further investigated. For *M. smegmatis* strains, Ms_rv0309 generated more rugose and thicker colonies than the Ms_Vec strain (**Figure 2C**, upper panel). For BCG strains, BCGΔRS01790 had an obviously thinner and less rugose colonies than WT BCG and cBCGΔRS01790::RS01790 strains (**Figure 2C**, lower panel) which were significantly larger and more like a circle compared with BCGΔRS01790 (**Figure 2D**). To further explore the alterations of colony size, we conducted an SEM image analysis on the bacterial size and the results showed that the proportion of bacteria with shorter length (3.5μm, the median of bacterial length of BCG strains) in the images of BCGΔRS01790 strain is 60%, while that of WT BCG only 10% showing significant difference between them (p<0.01) (**Figure 2E**). The widths did not differ among the three BCG strains. In addition, no difference in bacterial length and width was observed between Ms_rv0309 and Ms_Vec (**Figure 2E**). Furtherly, the growth curve was also determined, but showed no significant difference in neither *M. smegmatis* strains nor BCG strains (**Figure 2F**).

These results suggested that Rv0309 can increase the formation of Mycobacterium wrinkle and help BCG to grow bigger but didn’t influence the growth rate of Mycobacterium.

### Rv0309, as a Cell Wall Component, Reduces Cell Wall Permeability

**Figure 3:**
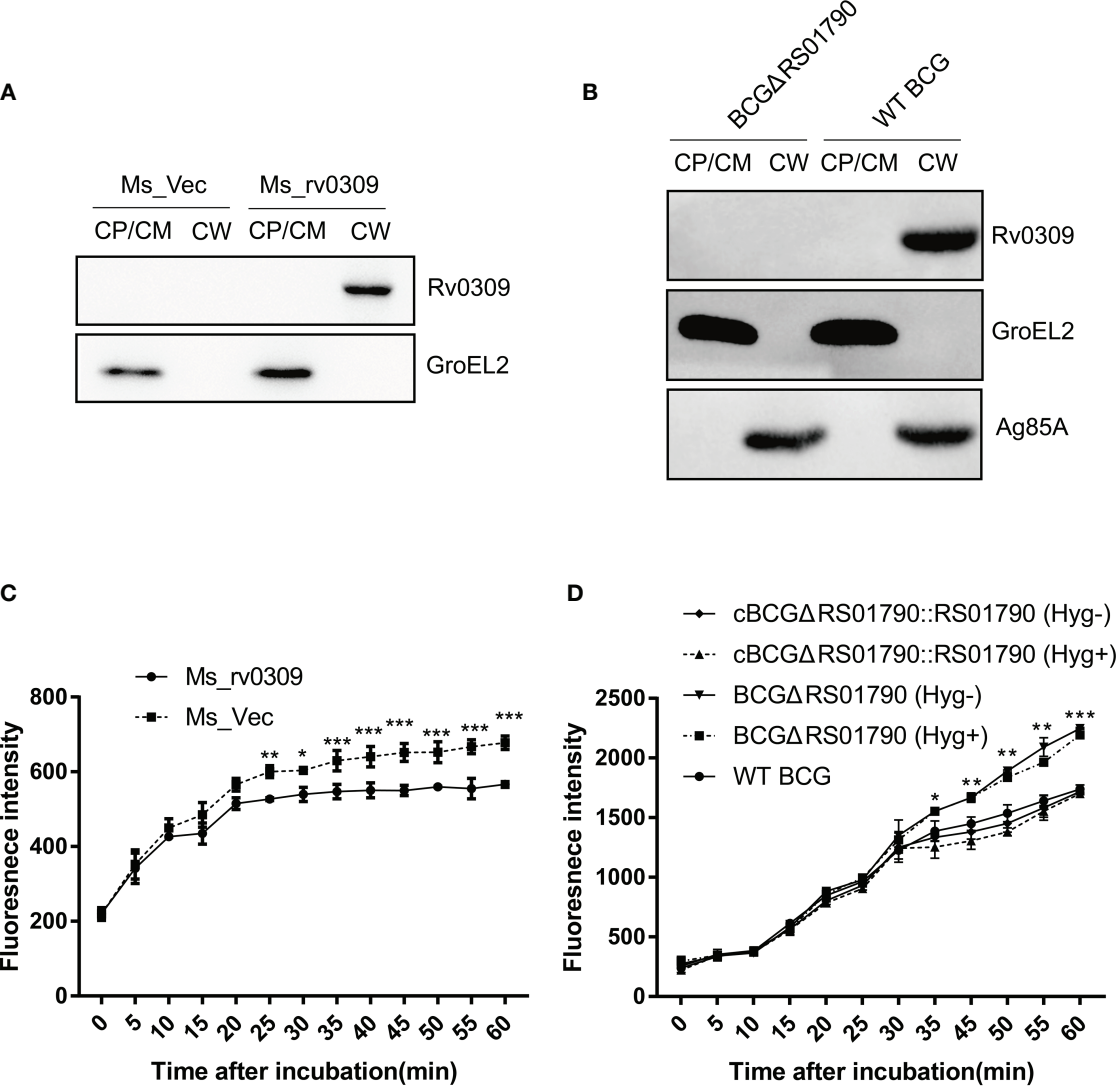
Subcellular localization of Rv0309 in mycobacteria and its effect of Rv0309 on the mycobacterial cell permeability. **(A, B)** Ms_rv0309, Ms_Vec, and WT BCG were cultured until an OD_600_ of 0.6. Various mycobacterial cell fractions were separated to determine the subcellular localization of Rv0309. GroEL2 was used as a cytoplasmic marker of *M. smegmatis* and BCG, while Ag85A as a cell wall marker of BCG. Western blotting assays were probed with Rv0309, GroEL2, or Ag85A antibodies to detect the target proteins. CW, cell wall; CM, cell membrane; CP, cytoplasm. **(C, D)** To examine the effect of Rv0309 on cell wall permeability, logarithmic growth phase Ms_Vec, Ms_rv0309, WT BCG, BCGΔRS01790, and cBCGΔRS01790::RS01790 cells were incubated in 7H9 containing 2 μM EB for the indicated periods. BCGΔRS01790 and cBCGΔRS01790::RS01790 were cultured with and without the antibiotic Hygromycin. Hyg+, with Hygromycin; Hyg-, without Hygromycin. A two-tailed unpaired *t*-test and two-way ANOVA were used to determine the statistical significance of differences between different groups (three independent replicates). *p < 0.05, **p < 0.01, and ***p < 0.001 indicate statistically significant differences.

To determine the subcellular location of Rv0309, Ms_rv0309, and WT BCG cells were disrupted by sonication, and cell wall and cell membrane/cytoplasmic fractions were extracted and analyzed by western blotting. Results showed that Rv0309/BCG_RS01790 protein was solely present in the cell wall of Ms_rv0309 and WT BCG, not in the cytoplasm or cell membrane (**Figures 3A, B**). As expected, the cytoplasmic protein GroEL2 of *M. smegmatis* and BCG, evaluated as a positive control, was detected in cytoplasm of *M. smegmatis* and BCG. As a positive control for cell wall fraction, Ag85A was detected only in the cell wall fraction of BCG.

To explore possible Rv0309-induced alterations in the cell wall architecture, a permeability assay was conducted. At 1 h after EB treatment, EB accumulation was significantly lower in Ms_rv0309 cells than in Ms_Vec cells (**Figure 3C**), and the intracellular EB accumulation of WT BCG and cBCGΔRS01790::RS01790 was significantly lower than BCGΔRS01790 (**Figure 3D**). These results indicated that Rv0309 can reduce cell wall permeability.

### Rv0309 Enhances Mycobacterial Intracellular Survival

**Figure 4:**
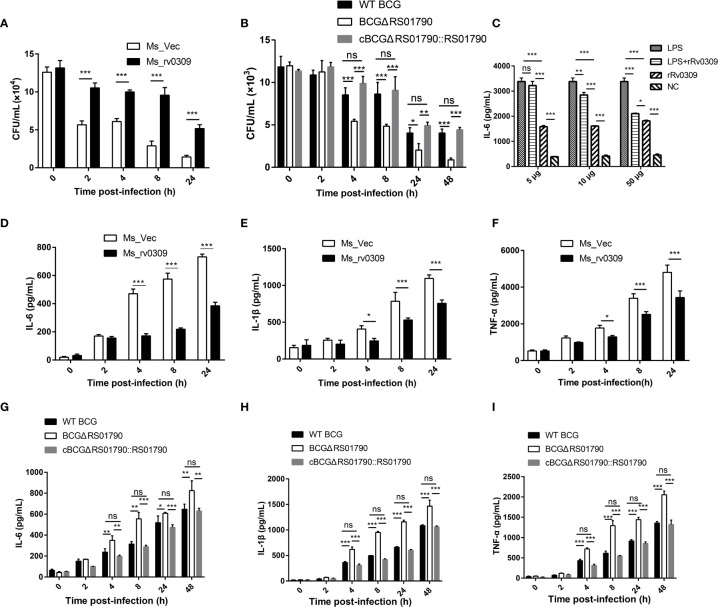
*M. tb* Rv0309 enhanced mycobacterial intracellular survival and inhibited pro-inflammatory cytokine production. **(A, B)** Numbers of intracellular mycobacterial cells, including Ms_Vec, Ms_rv0309 **(A)**, WT BCG, BCGΔRS01790, and cBCGΔRS01790::RS01790 **(B)**, were recorded at 0, 2, 4, 8, 24 and 48 hour post-infection (hpi). **(C)** IL-6 levels in supernatants of RAW264.7 cells treated with rRv0309 protein at 5, 10, and 50 μg/mL and LPS at 1 μg/mL were detected by ELISAs. **(D–I)** ELISAs were used to determine IL-6, IL-1β, and TNF-α levels in supernatants of RAW264.7 macrophages infected with Ms_Vec, Ms_rv0309 **(D–F)**, WT BCG, BCGΔRS01790, and cBCGΔRS01790::RS01790 **(G–I)** at 0, 2, 4, 8, 24 and 48 hpi. Two-way ANOVA was used to determine the statistical significance of differences between the treatments (three independent experiments). *p < 0.05, **p < 0.01, and ***p < 0.001 indicate statistically significant differences; while ns indicates no significant difference.

To confirm the role of Rv0309 in intracellular mycobacterial survival, RAW264.7 cells were infected with Ms_rv0309, Ms_Vec, BCGΔRS01790, WT BCG, and cBCGΔRS01790::RS01790 at an MOI of 10:1. A plate counting assay of intracellular mycobacteria showed that the intracellular bacterial amount (CFU/mL) was significantly higher for Ms_rv0309 than for Ms_Vec at 2, 4, 8, and 24 hpi (**Figure 4A**). Similarly, intracellular bacterial survival of BCGΔRS01790 at 4, 8, 24, and 48 hpi were significantly decreased, whereas that of cBCGΔRS01790::RS01790 was recovered to the levels observed for the WT BCG (**Figure 4B**). In addition, compared to 0 hpi, the amount of Ms_Vec (CFU/ml) sharply decreased by 55% at 2 hpi but was not completely cleared, maintained at a stable status through 4 hpi, then continuously decreased by 77% at 8 hpi and 89% at 24 hpi. Although the amount of MS_rv0309 decreased at a similar trend, it kept a slow and constant reduction within the first 8 hpi with the decreasing rate of 20%, 24% and 27% at 2, 4 and 8 hpi respectively, then a higher decreasing rate of 61% at 24 hpi. A similar effect of BCG_RS01790 on BCG survival was observed in BCG strains, but the significant decrease of BCG strains occurred at 4 hpi, 2 h behind of *M. smegmatis*. These data indicated that Rv0309/BCG_RS01790 significantly enhances the intracellular survival of *M. smegmatis* and BCG in macrophages.

### Rv0309 Inhibits Pro-Inflammatory Cytokine Production

To reveal the mechanism by which Rv0309 promotes intracellular bacterial survival, cytokines induced by LPS, and bacterially infected models were determined. Data showed that after induced by LPS, 10 μg/mL (*p* < 0.01) and 50 μg/mL (*p* < 0.001) rRv0309 can significantly decrease the production of IL-6 (**Figure 4C**). The same trend occurred in both *M. smegmatis*- and BCG- infected RAW264.7 cells. Cells infected with Ms_rv0309 exhibited significantly lower levels of IL-6 (**Figure 4D**). BCG_ΔRS01790 increased the production of IL-6 compared with WT BCG and cBCGΔRS01790::RS01790 (**Figure 4G**). Besides IL-6, Ms_rv0309 also exhibited the production of IL-1β and TNF-α from 4 hpi (*p* < 0.05) and 8 hpi (*p* < 0.001), respectively (**Figures 4E, F**). And BCGΔRS01790 increased the expression level of IL-1β and TNF-α from 4 hpi (*p* < 0.01) compared with WT BCG and complement strain cBCGΔRS01790::RS01790 (**Figures 4H, I**).

The transcription level of IL-6, IL-1β, TNF-α effected by Rv0309 in RAW264.7 cells was analyzed using RT-qPCR and were in agreement with the ELISA data (**Supplementary Figure 2**).

Taken together, these results indicated that Rv0309 can inhibit the production of pro-inflammatory cytokines including IL-6, IL-1β, and TNF-α.

### NF-κB p65/IκBα and MAPK JNK/ERK Signaling Are Critical for the Inhibition of Cytokine Production by Rv0309

**Figure 5:**
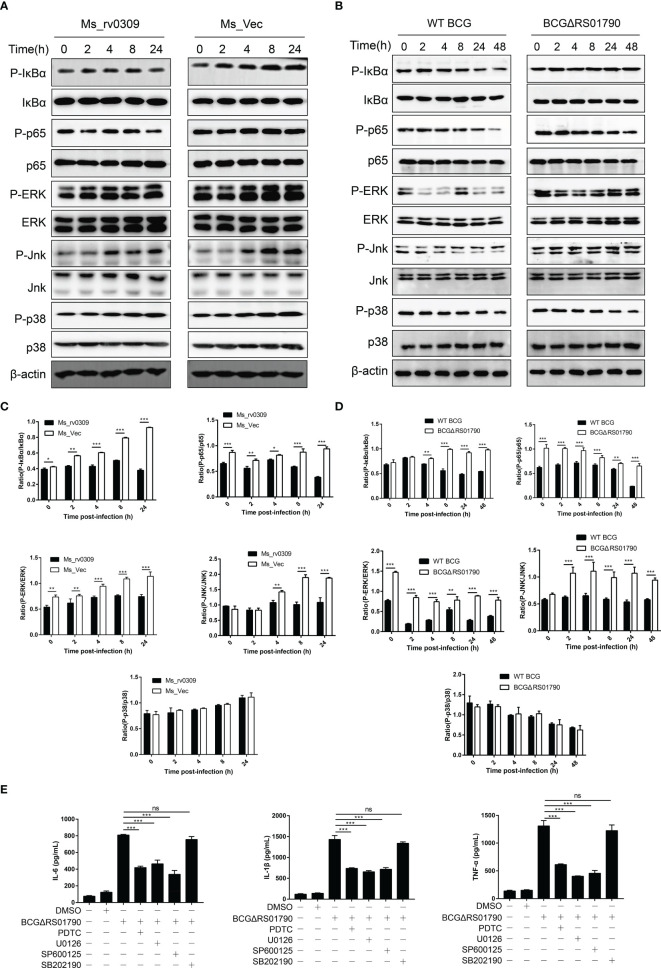
*M. tb* Rv0309 suppressed NF-κB, ERK, and JNK pathway activation. **(A, B)** Western blot analysis of phosphorylated and non-phosphorylated IκBα, p65, JNK, ERK, p38 in RAW264.7 cells with Ms_Vec, Ms_rv0309 **(A)**, WT BCG, BCGΔRS01790, and cBCGΔRS01790::RS01790 **(B)** at 0, 2, 4, 8, 24 and 48 hours post-infection (hpi). β-Actin was used as a loading control. **(C, D)** P-IκBα/IκBα, P-p65/p65, P-ERK/ERK, P-JNK/JNK, and P-p38/p38 ratios. **(E)** RAW264.7 cells were pretreated with PDTC (NF-κB inhibitor), 10 mM U0126 (EK1/2 inhibitor), 20 mM SP600125 (JNK inhibitor) and 20 mM SB202190 (p38 inhibitor) for 1 hour before being infected with BCGΔRS01790. The culture supernatants were collected at 12 h after infection, and the concentrations of IL-6, IL-1β, and TNF-α were determined. One-way and two-way ANOVA were used to determine the statistical significance of differences between the treatments (similar results were obtained in three independent experiments). *p < 0.05, **p < 0.01, and ***p < 0.001 indicate statistically significant differences; while ns indicates no significant difference.

To further elucidate the mechanism by which Rv0309 inhibits cytokine production, we examined critical signaling molecules, including MAPK JNK, MAPK ERK, MAPK p38, NF-κB p65, and IκBα in RAW264.7 cells infected with Ms_rv0309, Ms_Vec, WT BCG, and BCGΔRS01790 by western blotting. The phosphorylation of IκBα and NF-κB p65 (from 0 hpi onwards, i.e., the very beginning of the observation) was significantly inhibited in Ms_rv0309-infected cells compared to Ms_Vec-infected cells (**Figures 5A, C**). After Ms_rv0309 infection, in the MAPK signal pathway, the phosphorylation of ERK was decreased, and that of JNK was decreased at 4, 8, and 24 hpi (**Figures 5A, C**). However, p38 phosphorylation showed no significant difference between the Ms_Vec control group and Ms_rv0309 treatment group. Similar results were also obtained from the immunoblotting analysis of RAW264.7 cells infected with different BCG strains including BCGΔRS01790 and WT BCG. The phosphorylation of IκBα (from 4 hpi onwards) and NF-κB p65 (from 0 hpi onwards) were enhanced by BCGΔRS01790 infection compared to WT BCG infected cells (**Figures 5B, D**). In the MAPK signal pathway, the BCGΔRS01790 infection stimulated significantly increased phosphorylation of ERK and JNK (**Figures 5B, D**). However, the phosphorylation of p38 did not differ between BCGΔRS01790 and WT BCG-infected cells. To further confirm the above signaling molecules were engaged in Rv0309 action, RAW264.7 cells were pretreated with the inhibitors PDTC (NF-κB inhibitor), U0126 (ERK1/2 inhibitor), SP600125 (JNK inhibitor), and SB202190 (p38 inhibitor) for 1 hour before being infected with BCGΔRS01790. As a result, the inhibitors PDTC, U0126, and SP600125 treatment in BCGΔRS01790-infected cells exhibited significant inhibition of IL-6, IL-1β, and TNF-α production and release (**Figure 5E**). However, SB202190 did not display any significant inhibitory effect on the cytokine production in the RAW264.7 cells infected by BCGΔRS01790. Thus, Rv0309 regulates cytokine secretion mainly by inhibiting the phosphorylation of NF-κB p65/IκBα and MAPK JNK/ERK signaling pathways.

### Rv0309 Suppresses Innate Immune Responses and Exacerbates Lung Lesions and Bacterial Loads in Mice

**Figure 6:**
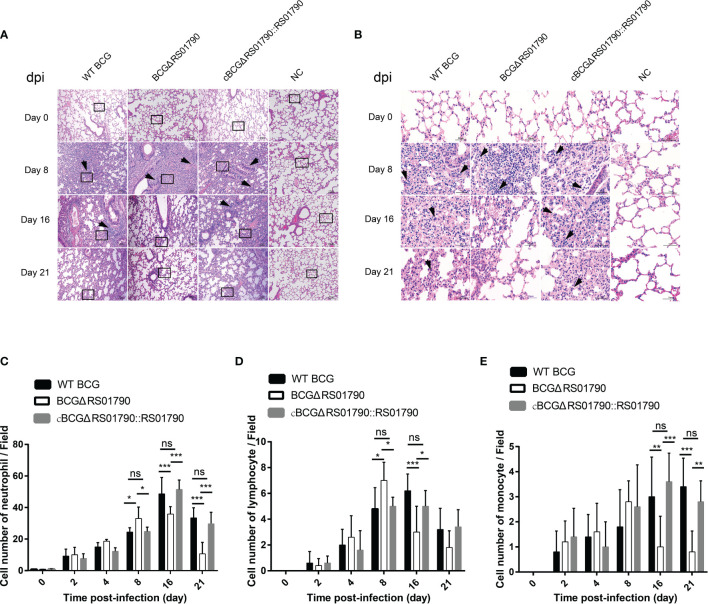
Deletion of Rv0309/BCG_RS01790 reduced lung inflammatory lesions in mice infected with BCG strains. **(A)** Mice were challenged with WT BCG, BCGΔRS01790, or cBCGΔRS01790::RS01790 at a dose of 2.0 × 10^6^ CFU by the intratracheal challenge at different times. The histopathology of mouse lungs was evaluated using hematoxylin and eosin-stained sections. **(B)** Enlarged views of the regions outlined in **(A)**. Arrows **(A, B)** pinpoint foci of cellular infiltration **(A)** and neutrophils (with a lobulated nucleus) **(B)**, day post-infection (dpi). **(C–E)** Quantitative analysis of neutrophils **(C)**, lymphocytes **(D)**, and monocytes **(E)** in each field. Two-way ANOVA was used to determine the statistical significance of differences between the treatments (n = 5 mice/group). *p < 0.05, **p < 0.01, and ***p < 0.001 indicate statistically significant differences; while ns indicates no significant difference.

The role of Rv0309 in suppressing inflammatory responses was further evaluated in C57BL/6 mice. In general, lung damage aggravated over time. WT BCG and cBCGΔRS01790::RS01790 caused more severe lung damage than BCGΔRS01790. Histopathological changes in lung tissues were observed as of 8 dpi and mainly manifested as immune cell infiltration, alveolar wall thickening, and the fusion of alveolar cavities, which ultimately led to the collapse of the lung tissue structure (**Figures 6A, B**). Most cells in the tissue sections were identified as neutrophils, and lymphocytes and monocytes were also identified. In the early stage (8 dpi), neutrophils and lymphocytes were significantly more abundant in mice infected with BCGΔRS01790 than in mice infected with the other two strains (**Figures 6C, D**) (*p* < 0.05), whereas in the late stage of the experiment, opposite trends were observed. In addition, the number of monocytes remained low in the early stage but increased in the late stage of the experiment (16 and 21 dpi). Similarly, monocyte numbers were significantly lower in the BCGΔRS01790 infection group than in the other two infection groups at both 16 and 21 dpi (**Figure 6E**).

**Figure 7:**
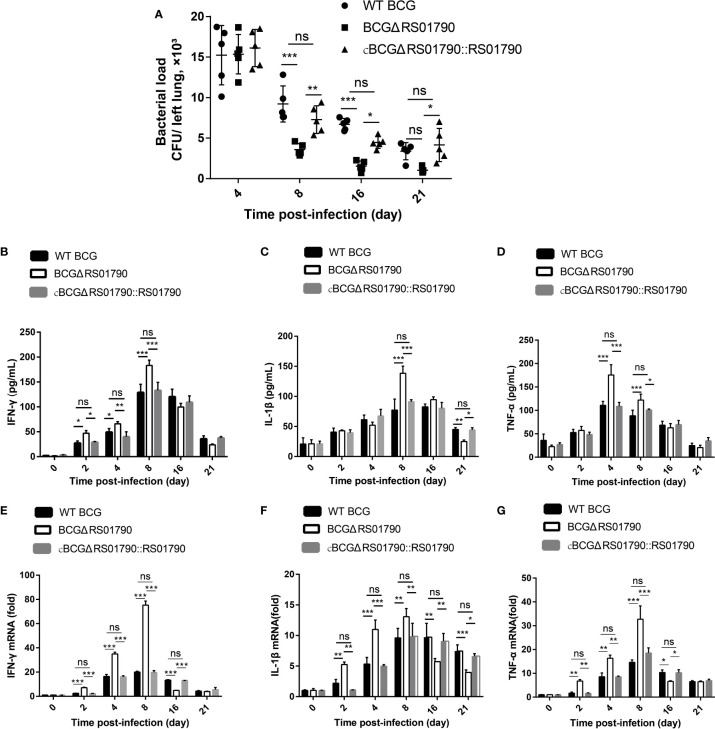
Rv0309/BCG_RS01790 suppressed host innate immune responses induced by mycobacterial infection. **(A)** Bacterial counts in lung homogenates from mice challenged intratracheally with WT BCG, BCGΔRS01790, and cBCGΔRS01790::RS01790 at 2.0 × 10^6^ CFU/mouse for 0–21 days. **(B–D)** ELISAs were used to quantify serum levels of IFN-γ **(B)**, IL-1β **(C)**, and TNF-α **(D)** in mice infected as in **(A)**. **(E–G)** RT-qPCR analysis of mRNA levels of IFN-γ **(E)**, IL-1β **(F)**, and TNF-α **(G)** in splenic cells from mice treated as in **(A)**. Two-way ANOVA was used to determine the statistical significance of differences between the treatments (n = 5 mice/group). *p < 0.05, **p < 0.01, and ***p < 0.001 indicate statistically significant differences; while ns indicates no significant difference.

Next, lung bacterial loads were examined and the bacterial loads of all three strains were significantly decreased at 8 dpi. Notably, in BCGΔRS01790-infected mice, the lung bacterial loads sharply decreased and reached the minimum levels at 8 and 16 dpi compared with that in mice infected with WT BCG or cBCGΔRS01790::RS01790 (**Figure 7A**).

Cytokine production was examined at both the mRNA and protein levels. In general, the BCGΔRS01790 infection group exhibited significantly higher serum levels of IFN-γ, TNF-α, and IL-1β than WT BCG and cBCGΔRS01790::RS01790 groups before 8 dpi. However, after 8 dpi, the cytokine concentrations of IFN-γ, TNF-α, and IL-1β in BCGΔRS01790 infection group were lower or similar compared with the other two infection groups at one or more time points (**Figures 7B–G**).

**Figure 8:**
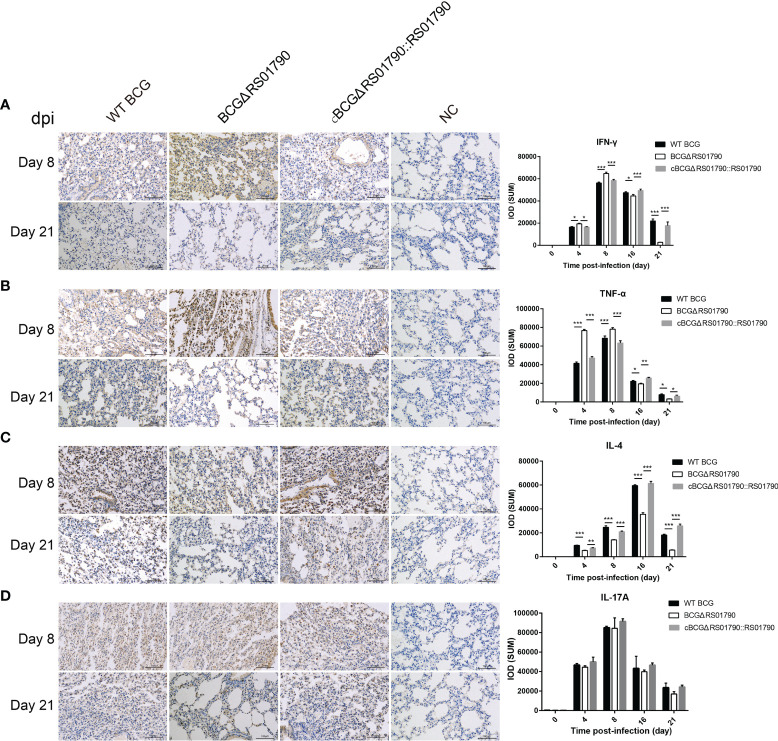
Detection of lung inflammatory cytokines induced by various strains with or without Rv0309/BCG_RS01790 using immunohistochemistry. **(A–D)** Immunohistochemical detection of the cytokines IFN-γ **(A)**, TNF-α **(B)**, IL-4 **(C)**, and IL-17A **(D)** in the lungs of mice infected with WT BCG, BCGΔRS01790, or cBCGΔRS01790::RS01790 at the indicated days post-infection. Two-way ANOVA was used to determine the statistical significance of differences between the treatments (three independent experiments). *p < 0.05, **p < 0.01, and ***p < 0.001 indicate statistically significant differences.

Immunohistological analysis of lung tissues revealed that the levels of IFN-γ and TNF-α, which are representative Th1 cytokines, were significantly higher in the BCGΔRS01790 infection group than in the WT BCG and cBCGΔRS01790::RS01790 infection groups at 4 and 8 dpi, and they were significantly lower after 16 dpi (**Figures 8A, B**). The level of IL-4, a Th2 hallmark cytokine, was significantly lower in the BCGΔRS01790 infection than in the other two groups throughout the infection period (**Figure 8C**). The IL-17A level was significantly increased in all three infection groups compared to the mock-infected group, without a significant difference among the infection groups (**Figure 8D**).

In addition, we examined the body and organ weights of the mice after infection. Compared with mice infected with WT BCG or cBCGΔRS01790::RS01790, mice infected with BCGΔRS01790 gained significantly more weight in late stage of the experiment. (**Supplementary Figure 3A**) (*p* < 0.01). After the mice were euthanized, we recorded the weights of the lungs, spleens, and livers and calculated the organ-to-body weight ratios. We observed no differences in the ratios among the bacterial infection and mock infection groups (*p* > 0.05) (**Supplementary Figures 3B**–**D**).

## Discussion

Innate immunity, including the inflammatory response, represents the first line of host defense against pathogen infection (43). However, pathogens can suppress the inflammatory response in the host to ensure their survival. This two-way process likely is very complex, and little is known about the mechanism by which intracellular bacteria such as *M. tb* commonly infecting macrophages can persist in a latent state throughout the life of the host. In this study, we revealed a new putative conservative *M. tb* protein Rv0309, which can elicit a better bacterial survival ability *in vivo* and *in vitro* and inhibit the inflammatory immunity by suppressing NF-κB and MAPK JNK/ERK signaling pathway. Moreover, it can help the bacterium lead to severe lung damage and diffuse inflammation in mice.

Virulence of mycobacterium was associated with many factors, including rough morphology, intracellular survival, cell wall permeability, cell envelope proteins, and so on (23, 44). In this study, Rv0309 was predicted to contain a YkuD_like superfamily domain with an L,D-transpeptidase catalytic site, which is related to the hinge of bacterial cell wall peptidoglycans and is the target of β-lactam drugs. In *M. tb*, peptidoglycan is cross-linked mainly by L,D-transpeptidases, which can be efficiently inactivated by a single β-lactam class, carbapenems and was reported essential for *M. tb* survival during chronic infection of mice (45, 46). Here we didn’t verify the L,D-transpeptidase activity in this study, but the phenomena that Rv0309 decreased the cell wall permeability in both *M. smegmatis* and BCG, while making the mycobacterium colony rough, were all agree with the YkuD_like superfamily domain’s function, together with the cellular location and cytoplasmic fractions results, we confirmed that Rv0309 is localized in the mycobacterial cell wall and involved in the formation of the cell wall structure, which can enhance the virulence of *M. tb* (47). Expression of Rv0309 enhanced the intracellular survival of *M. smegmatis* and BCG *in vivo* and *in vitro*. Here we found that, at 2 h post-infection, more Ms_rv0309 cells than Ms_Vec cells survived in macrophages. And in the mouse experiment, the lung bacterial load of mice infected with the BCGΔRS01790 strain was significantly lower than that of mice infected with WT BCG at 8 dpi. This might be partially attributed to the YkuD_like superfamily domain with the L,D-transpeptidase catalytic site which decreases the permeability of cell wall, then increase mycobacterial resistance to environmental stresses and thereby intracellular survival. However, this needs further investigation.

The characteristic response to primary infection with *M. tb* involves the localized accumulation of mixed inflammatory cells (48). In our current study, all BCG inoculated mice had an accumulation of inflammatory cells, as the disease progressed, both WT BCG and cBCGΔRS01790::RS01790 strains induced significantly higher amounts of neutrophils, lymphocytes, and monocytes than BCG∆RS01790-infected mice and cBCGΔRS01790::RS01790 strain infected mice exhibited a much more severe inflammatory changes. According to the previous studies, macrophages can be functionally polarized to M1 or M2 phenotypes in response to different microbial infections (49). In the current study, Rv0309 protein inhibited the production of M1-related cytokines (IL-1β and TNF-α) and lead to the promotion of bacterial survival in the *in vitro* experiment. As previously reported, TNF-α can promote the inhibition and/or killing of *M. tb* in the host (50–52), inducing autophagy, promoting the fusion of *M. tb* phagosomes with autophagosomes, eventually facilitating bacilli clearance from autophagolysosomes (53, 54). IL-6 is critical for protective immune response activation and mycobacterial killing (55–57). IL-1β has paradoxical effects on the control of pro-inflammatory pathways. On one hand, pro-inflammatory pathways can inhibit bacterial replication to prevent disease, while on the other hand, they can promote disease by causing excessive inflammation damage (58, 59). This was agreed with previous studies. *M. tb* Rv2346c has been reported to improve the survival of mycobacteria by impeding TNF-α and IL-6 production through the p38/miRNA/NF-κB pathway in macrophages (50). PtpA can decrease TNF-α and IL-1β expression and enhanced the lung bacterial load in mice challenged with BCG (6). Therefore, the inhibitory effect of Rv0309 on cytokine production likely is responsible for improved mycobacterial survival.

For the *in vivo* studies, mutant strain BCGΔRS01790 strain caused a higher level of M1-related Th1 cytokines (IFN-γ, IL-1β, and TNF-α) secretion around 8 and 16 dpi, respectively, which may support that Rv0309 protein may inhibit macrophage M1 polarization, and conducive to the intracellular survival of mycobacteria at the early stage of infection. This inhibitory effect of Rv0309 may further suppress Th1-acquired immune responses. The immunohistological assay on the tissues and ELISA on serum cytokines confirmed the hypothesis. BCGΔRS01790 infection increased the levels of Th1 cytokines IFN-γ, IL-1β, and TNF-α in the early stage of infection (at 8 dpi), followed by a sharp decrease in the late stage of the experiment (at 16 dpi), whereas no significant differences in Th17 hallmark cytokines. As the infection progressed (at day 16 and 21 post infection), the infected cells likely cleared more BCGΔRS01790 which was confirmed by the less mycobacterial lung load. From day 8 on post-infection, the mycobacterial lung load in BCGΔRS01790-infected mice dropped mostly compared to another two groups likely resulting from the higher expression levels of Th1-related cytokines, better M1 macrophage polarization and more clearance of BCGΔRS01790. In turn, a decrease in the mycobacterial lung load led to the less stimulation of immune cells including macrophages and thereby lower production of Th1 cytokines levels and suppression of Th1-acquired immune responses.

Further data showed that phosphorylation levels of MAPK ERK, MAPK JNK, NF-κB IκBα, and NF-κB P65 were significantly lower in Ms_rv0309- and WT BCG- infected macrophages than in Ms_Vec- and BCGΔRS01790-infected macrophages, which indicated that the alterations in IL-6, TNF-α, and IL-1β expression were affected by MAPK JNK and MAPK ERK and NF-κB activation in macrophages. This was agreed with the previous studies. *M. tb* Mce3E can inhibit the JNK and ERK signaling pathways, thus suppressing IL-6 and TNF-α expression and promoting mycobacterial survival within macrophages (60, 61). Although the MAPK and NF-κB pathways are distinct signaling pathways, they share parts when activated *via* Toll-like receptors (TLRs) (62). Further, the Rv0309 protein has been identified as a novel adhesin of *M. tb* H37Rv, and it can bind to fibronectin and laminin (20). Fibronectin can interact with integrin β1 in macrophages to activate TLR2- and TLR4-related signaling pathways, thus enhancing the expression of pro-inflammatory mediators and phagocytosis by macrophages (63–66). Therefore, it can be speculated that Rv0309 may be involved in the co-activation of the MAPK and NF-κB signaling pathways through interaction with TLR receptors. However, this has to be further explored in the future.

**Figure 9:**
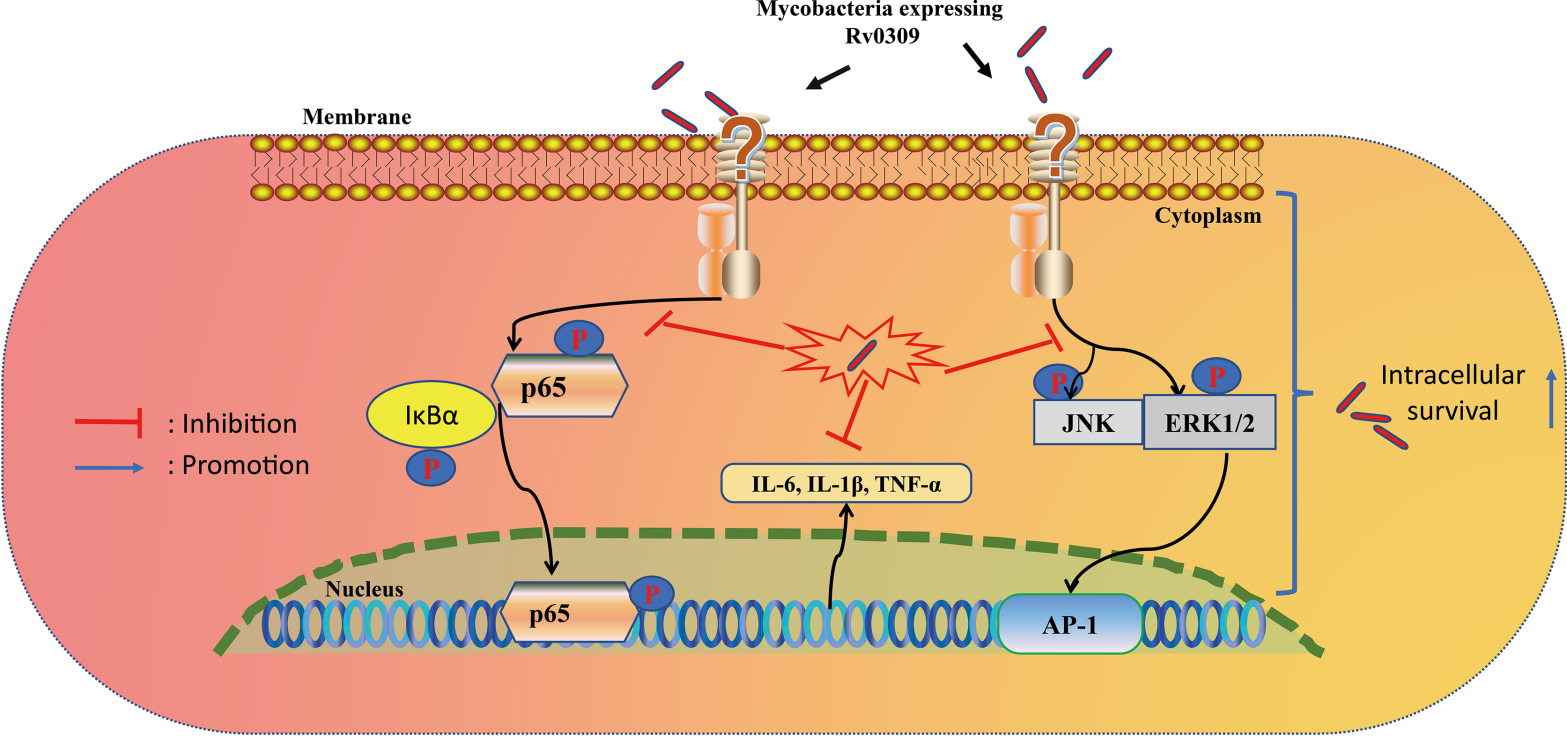
Proposed model of Rv0309 interaction with macrophages. Rv0309 enhances intracellular mycobacterial survival by inhibiting proinflammatory cytokine expression through suppression of phosphorylation of NF-κB, MAPK JNK/ERK 1/2 signaling pathways.

In summary, this study revealed a new putative cell wall protein Rv0309/BCG_RS01790, which can affect the mycobacterium colony morphology, reduce the permeability of bacterial cell wall, promote bacterial survival in host cells, more importantly, suppress the production of pro-inflammatory cytokines through NF-κB, MAPK ERK, and MAPK JNK signaling pathways, and help mycobacterium to enhance lung damage of mice (**Figure 9**). Therefore, this cell wall protein Rv0309/BCG_RS10790 is a novel virulence-related factor and might be a potential drug target for *M. tb* treatment. In addition, this provides new insights into *M. tb* persistence and lays a solid foundation for the development of effective prophylactic and therapeutic strategies to combat tuberculosis.

## Data Availability Statement

The original contributions presented in the study are included in the article/**Supplementary Material**. Further inquiries can be directed to the corresponding authors.

## Ethics Statement

The animal study was reviewed and approved by the institutional ethics committee on animal experimentation of the Laboratory Animal Center of Huazhong Agricultural University (permitted protocol no: HZAUMO-2018-027).

## Author Contributions

AG and YC contributed to the conception and design of the study. YP, XZ, and LG carried out the experiment and wrote sections of the manuscript. YZ, HL, XC, and TZ were involved in Bacterial and cell culture studies. XT, CH, HC, and JW performed the statistical analysis. YP, AG, and YC wrote the manuscript with all authors providing feedback. All authors contributed to manuscript revision, proof-reading, and approval of the submitted version.

## Funding

This work was supported by the National Natural Science Foundation of China (32072942), the National Key Research and Development Program of China (SQ2021YFD1800012), Natural Science Foundation of Hubei Province (2021CFA016), and China Agriculture Research System of MOF and MARA (CARS-37).

## Conflict of Interest

The authors declare that the research was conducted in the absence of any commercial or financial relationships that could be construed as a potential conflict of interest.

## Publisher’s Note

All claims expressed in this article are solely those of the authors and do not necessarily represent those of their affiliated organizations, or those of the publisher, the editors and the reviewers. Any product that may be evaluated in this article, or claim that may be made by its manufacturer, is not guaranteed or endorsed by the publisher.

## Supplementary Material

Supplementary Figure 1 Confirmation of *rv0309/*BCG_RS01790 insertion in Ms_rv0309 and deletion in BCGΔRS01790 by PCR. **(A)** The rv0309 gene from the recombinant strain Ms_rv0309 was PCR-amplified using specifically designed primers. The PCR product has the expected size (657 bp). **(B)** The deletion-substitution mutant BCGΔrv0309 was confirmed using PCR primers RS01790LYZ and RS01790RYZ. The products with the expected sizes were obtained for the WT (~2.5 kb) and knockout (~5.5 kb) strains. Lane M, 2kb and 15kb DNA ladder; lane NC, negative control.

Supplementary Figure 2 Assays on cytokine gene expression in RAW264.7 cells infected with various strains with or without Rv0309. **(A-F)** RT-qPCR analysis on mRNA levels of *IL-6*, *TNF-α*, and *IL-1β* in RAW264.7 macrophages infected with Ms_Vec, Ms_rv0309, WT BCG, BCGΔRS01790, or cBCGΔRS01790::RS01790 at 0, 2, 4, 8, 24, and 48 hpi. Target mRNA levels are presented relative to those of the β-actin gene. Two-way ANOVA was used to determine the statistical significance of differences between the treatments (three independent experiments).

Supplementary Figure 3 Lung, liver, spleen, and body weights of C57BL/6 mice infected with WT BCG, BCGΔRS01790, or cBCGΔRS01790::RS01790. **(A)** The body weights of the mice were recorded before euthanasia. **(B–D)** Lungs **(B)**, livers **(C)**, and spleens **(D)** of mice after euthanasia were harvested and weighed, and the organ-to-body weight ratios were calculated. Two-way ANOVA was used to determine the statistical significance of differences between the treatments (n = 5 mice/group).
