# Supplementary figures and images for: Mycobacterium tuberculosis Rv0309 Dampens the Inflammatory Response and Enhances Mycobacterial Survival
Source: Front Immunol. 2022 Feb 24;13:829410. doi: 10.3389/fimmu.2022.829410 (PMC8907127; doi:10.3389/fimmu.2022.829410)

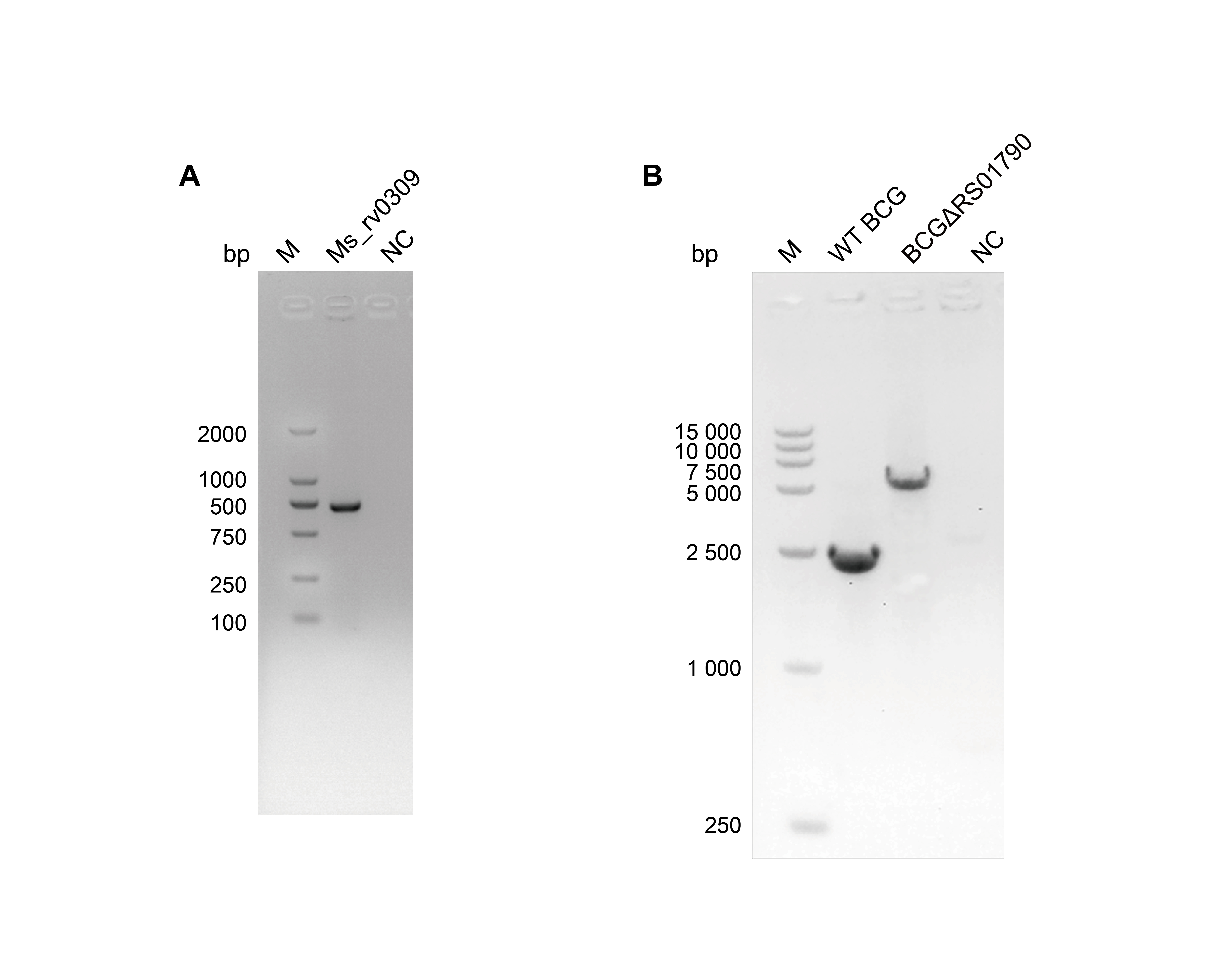

Supplement: Supplementary Figure 1 — Confirmation of rv0309/BCG_RS01790 insertion in Ms_rv0309 and deletion in BCGΔRS01790 by PCR. (A) The rv0309 gene from the recombinant strain Ms_rv0309 was PCR-amplified using specifically designed primers. The PCR product has the expected size (657 bp). (B) The deletion-substitution mutant BCGΔrv0309 was confirmed using PCR primers RS01790LYZ and RS01790RYZ. The products with the expected sizes were obtained for the WT (~2.5 kb) and knockout (~5.5 kb) strains. Lane M, 2kb and 15kb DNA ladder; lane NC, negative control. [file Image_1.jpeg]

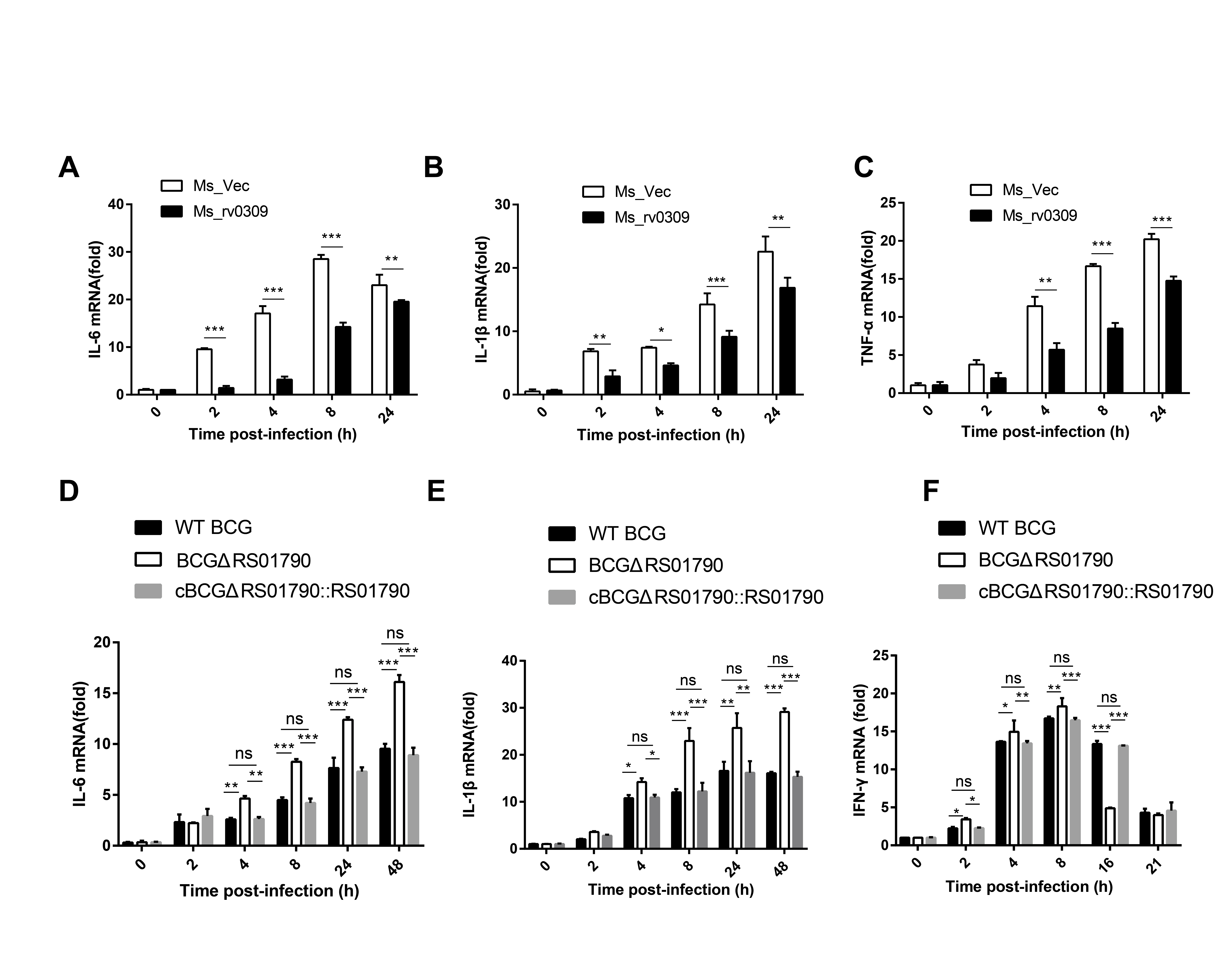

Supplement: Supplementary Figure 2 — Assays on cytokine gene expression in RAW264.7 cells infected with various strains with or without Rv0309. (A-F) RT-qPCR analysis on mRNA levels of IL-6, TNF-α, and IL-1β in RAW264.7 macrophages infected with Ms_Vec, Ms_rv0309, WT BCG, BCGΔRS01790, or cBCGΔRS01790::RS01790 at 0, 2, 4, 8, 24, and 48 hpi. Target mRNA levels are presented relative to those of the β-actin gene. Two-way ANOVA was used to determine the statistical significance of differences between the treatments (three independent experiments). [file Image_2.jpeg]

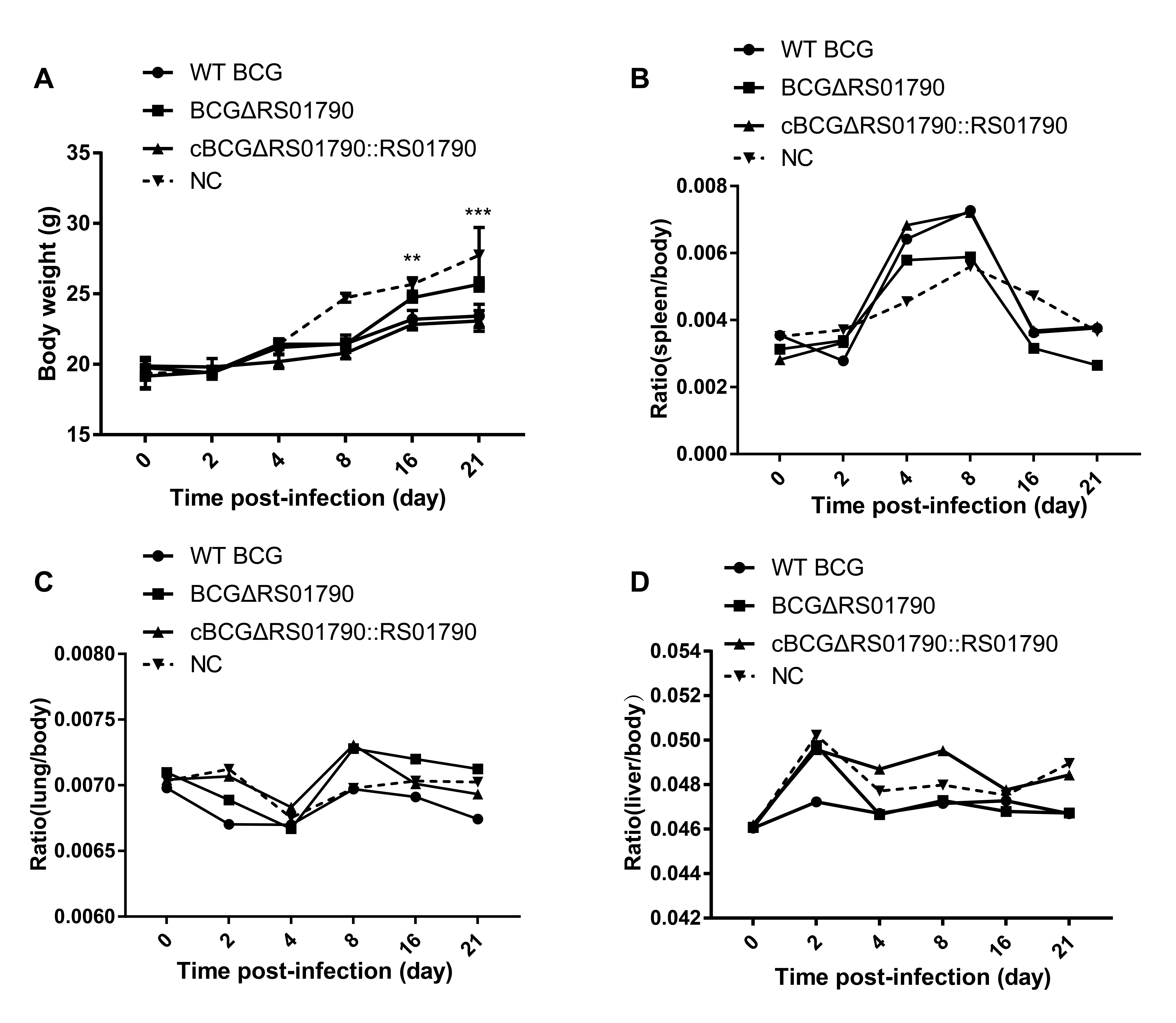

Supplement: Supplementary Figure 3 — Lung, liver, spleen, and body weights of C57BL/6 mice infected with WT BCG, BCGΔRS01790, or cBCGΔRS01790::RS01790. (A) The body weights of the mice were recorded before euthanasia. (B–D) Lungs (B), livers (C), and spleens (D) of mice after euthanasia were harvested and weighed, and the organ-to-body weight ratios were calculated. Two-way ANOVA was used to determine the statistical significance of differences between the treatments (n = 5 mice/group). [file Image_3.jpeg]
